# Integrative taxonomy reveals two new species of *Itajahya* Möller (Phallaceae, Phallales) in Brazil

**DOI:** 10.1371/journal.pone.0351926

**Published:** 2026-08-05

**Authors:** Mateus Santana Ribeiro, Tiara Sousa Cabral, Gislaine Cristina de Souza Melanda, Alessandra Selbach Schnadelbach, Bianca Denise Barbosa da Silva

**Affiliations:** 1 Programa de Pós–Graduação em Biodiversidade e Evolução, Instituto de Biologia, Universidade Federal da Bahia, Salvador, Bahia, Brazil; 2 Laboratório de Sistemática de Fungos, Instituto de Biologia, Universidade Federal da Bahia, Salvador, Bahia, Brazil; 3 Coordenação de Tecnologia e Inovação (COTEI), Instituto Nacional de Pesquisa do Amazonas, Manaus, Amazonas, Brazil; 4 Secretaria do Estado de Educação do Mato Grosso do Sul, Campo Grande, Mato Grosso do Sul, Brazil; 5 Laboratório de Genética e Evolução Vegetal, Instituto de Biologia, Universidade Federal da Bahia, Salvador, Bahia, Brazil; CONICET: Consejo Nacional de Investigaciones Cientificas y Tecnicas, ARGENTINA

## Abstract

*Itajahya* is a genus of phalloid fungi distinguished by a calyptra and a lamellar receptacle. Although historically treated as a synonym of *Phallus*, phylogenetic evidence supports its recognition as a distinct genus. In Brazil species delimitation within *Itajahya* has been based mainly on morphological traits, raising the possibility of overlooked diversity. Here, we investigated the diversity of *Itajahya* in Brazil using an integrative taxonomic approach combining morphological analyses and phylogenetic inference based on ITS, nucLSU, and *atp6*. Specimens were collected in the Chapada Diamantina region, northeastern Brazil. Bayesian and Maximum Likelihood analyses recovered *Itajahya* as a well–supported monophyletic lineage, clearly separating it from *Phallus*. Two new species, *Itajahya mucugensis* and *I. payaya* are described, raising from eight the number of species accepted in the genus. Both species form a well-supported clade sister which *I. rosea* and are distinguished from all previously described taxa by a combination of morphological and molecular characters. These findings reveal previously unrecognized diversity within *Itajahya* and emphasize the importance of integrative taxonomy for resolving species boundaries in phalloid fungi.

## Introduction

The genus *Itajahya* Möller belongs to the family Phallaceae and it is a phalloid fungi genus that was first described to science 130 years ago, in 1895, by the German mycologist and botanist Alfred Möller. The type species, *Itajahya galericulata* Möller, was originally described for the Blumenau region, in the state of Santa Catarina, Brazil [1]. Later, in 1927, the species *Alboffiella argentina* Speg. described in 1898 by the mycologist Carlos Luis Spegazzini, was transferred to the genus *Itajahya* by the same author and renamed *Itajahya argentina* (Speg.) Speg. [2].

In 1813, the French botanist Alire Raffeneau Delile described *Phallus roseus* Delile for Egypt [3] which was later recombined in *Itajahya rosea* (Delile) E. Fisch. by the botanist and mycologist Eduard Fischer in 1929 [4]. In 1954 *Itajahya hornseyi* Hansf. was described for Australia [5], bringing the number of accepted species in the genus to four.

After that, Kreisel synonymized *Itajahya* with *Phallus* Junius ex L., creating the subgenus of the same name, *Phallus* subgen. *Itajahya* (Möller) Kreisel [6]. This subgenus was morphologically defined by “pileus campanulate or wig–like, with flat and edged apex, imperforate. No indusium. Receptaculum without pinkish or without pigments” [6].

Despite this synonymizing, the taxa within *Itajahya* remained distinct at the species level. Consequently, *Phallus* subgen. *Itajahya* started to include the species *Phallus galericulatus* [6] Kreisel and *P*. *roseus* Delile, previously species of *Itajahya*, as well as *P*. *glutinolens* [6] O. Kuntze, originally described as *Ithyphallus* Gray, a taxon unrelated to *Itajahya*.

The genus *Itajahya* was reinstated in 2012 by Cabral and collaborators [7], who provided a phylogenetically positioning based on nucLSU and *atp6* sequences from a specimen of *Itajahya rosea* collected in Brazil. Recently, in 2025, two new species of *Itajahya* were described for Pakistan, supported by morphological and ITS–based molecular data: *Itajahya pakistanica* Bibi, Afshan & Khalid and *I*. *punjabensis* Bibi, Afshan & Khalid [8]

The main characteristic of the genus *Itajahya* is the presence of a membranous structure in the apical part of its receptacle, known as the calyptra, which distinguishes it from *Phallus* species without indusium. In addition to the calyptra, *Itajahya* can be differentiated from *Phallus* by looking at the nature of *Itajahya* species possess a lamellar receptacle, whereas *Phallus* species have membranous receptacles [1,5,9].

The species of *Itajahya* exhibit ecologically important interactions within their environments. Their spores are part of the diet of insects [10], suggesting an important role in spore dispersal. In addition, immature basidiomata of *I. galericulata* have been recorded in the diet of the rodents of the species *Dasyprocta azarae* Lichtenstein 1823, in Brazil [11]. With regard to interactions with plants, there are records of the association of *I. galericulata* with trees of the species *Jacaranda mimosifolia* D. Don in South Africa [12], and of *I. rosea* basidiomata reported occuring near of the root system of *Leucena leucocephala* (Lam.) de Wit, in India [10]. To date, however, no evidence of plant–associated interactions involving *Itajahya* species has been reported from Brazil.

Of the six currently accepted species of *Itajahya*, two are known to occur in Brazil: *Itajahya galericulata* and *I*. *rosea*. These species are distributed in the northeast, southeast and south regions of the country. *Itajahya galericulata* has been recorded in the states of Bahia [13], Paraíba [1], Rio de Janeiro [1], Rio Grande do Norte [14], Rio Grande do Sul [15,16] and Santa Catarina [16], while *I. rosea* has been reported in the states of Ceará [14] and Rio Grande do Norte [17]. Their occurrences are restricted to the Caatinga and Atlantic Rainforest biomes, with no records from the Amazon, Cerrado, Pampa and Pantanal biomes.

The color of the pseudostipe has been used as the main characteristic to distinguish *Itajahya* species, with white being attributed to *I*. *galericulata* and pink to *I*. *rosea*. However, literature records indicate the occurrence of intermediate colors, such as beige, yellow, and orange. Recent studies have shown that species of Phallales previously considered to be widely distributed actually correspond to taxonomic complexes [18–20].

Based on the above, the aim of this work is to investigate the diversity of *Itajahya* in Brazil, with the proposal of two new species based on an integrated approach combining morphological and molecular data.

## Materials and methods

### Specimen collection, morphological analyses, and loans

The specimens were collected in 2018 and 2024 in the Chapada Diamantina region, in the municipalities of Mucugê (13°00’26.63”S 41°22’12.47”W) and Morro do Chapéu (11°32’57.23”S 41°09’26;29W), Bahia, Brazil (Fig 1). Located within the Serra do Espinhaço, Brazil’s only mountain range, the Chapada Diamantina features the semi-arid climate characteristic of the Caatinga. The municipality of Mucugê is particularly characterized by the predominance of rupestrian fields, environments found at high altitudes (900 meters above sea level or higher), characterized by herbaceous vegetation and quatiziric soils [21]. The collections were carried out by actively searching for basidiomata in the field. The specimens were not collected in protected areas, so it was not necessary to obtain a permit for the fieldwork. Mature basidiomata were photographed in the field, while immature basidiomata were carefully removed with their substrate and transported to the laboratory, where they were incubated in humid chambers at room temperature until the peridium ruptured. When possible, immature basidiomata in an advanced stage of development were split longitudinally to observe the organization and composition of the peridium and its layers.

**Fig 1:**
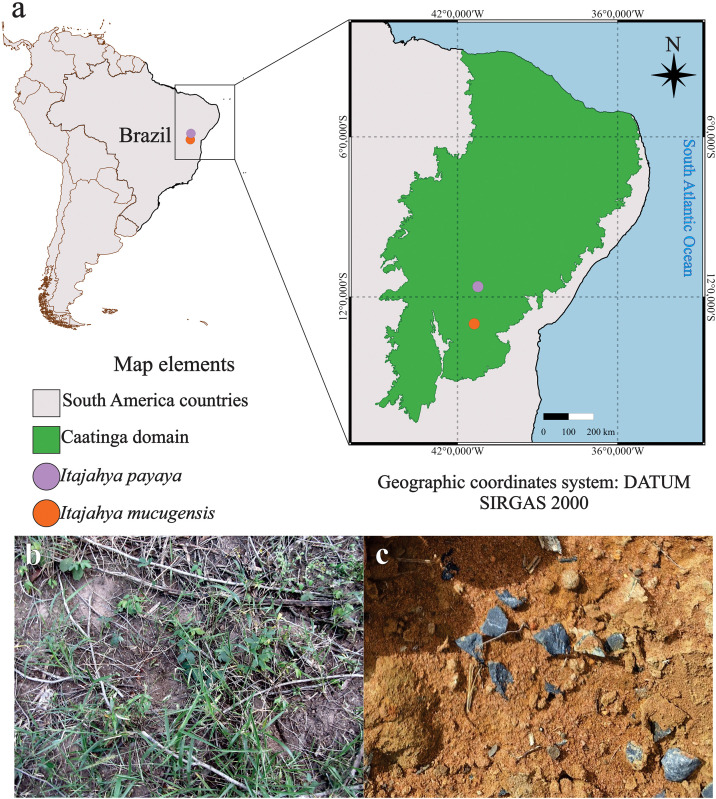
Map showing the collection sites of the species proposed as new in this study. **a.** South America countries, with detail of brazillian northeast showing the Caatinga Domain and collection points, being purple *Itajahya payaya* sp. nov. collected in the Morro do Chapéu city, and orange *Itajahya mucugensis* sp. nov., collected in Mucugê city. **b.** Soil in the site collection of *Itajahya mucugensis*. **c.** Soil in the site collection of *Itajahya payaya*. Map made with Datum SIRGAS 2000, public domain.

The measurements of the macroscopic structures (immature basidiomata, volva, pseudostipe, receptacle, and calyptra) were obtained using a digital caliper. After measurements, the basidiomata were dehydrated in a food dehydrator until completely dry. In addition, a duplicate of the ALCB141144 herbarium specimen was preserved in a solution of formaldehyde, alcohol, and glacial acetic acid (FAA). Preserving duplicates in FAA is advantageous, since the peridium layers and receptacle lamellae can be more easily separated for the preparation of microscope slides. All colors mentioned in the description of the macromorphology of the basidiomes are followed by their corresponding code in Küppers’ color chart [22].

Micromorphological observations were made from hand sections mounted in 5% potassium hydroxide (KOH) solution and subsequently stained with 1% Congo red. Slides were prepared from the following parts: base, middle, and apex of the exoperidium (20 cells from each part); mesoperidium and endoperidium; base, middle and expanded apex of the pseudostipe (20 cells from each part); calyptra; gleba (basidiospores and basidia); and lamellae of the receptacle. For each structure described, 20 measurements were taken. The observations and measurements were performed using a light microscope equipped with a digital camera, and image acquisition and measurement were conducted with Mosaic V2.4 software.

Additionally, to complement the molecular studies, herbarium material corresponding to the type locality of *Itajahya galericulata* was obtained on loan from the Dr. Roberto Miguel Klein Herbarium (FURB). The basidiomata collected from Mucugê and Morro do Chapéu were incorporated into the fungus collection of the Professor Alexandre Leal Costa Herbarium (ALCB), located at the Universidade Federal da Bahia, Brazil. Additionally, duplicates were incorporated into the fungus collection of the Universidade Federal do Rio Grande do Norte Herbarium.

### DNA extraction, PCR amplification and sequencing

DNA extraction was performed by adapting the DNA extraction protocol available in Cabral [23]. Fragments of the pseudostipe and gleba were frozen with liquid nitrogen and macerated until a fine powder was obtained. The maceration product was treated with 500 µL of CTAB for cell lysis and then incubated at 60°C for 30 minutes.

After incubation, the solution was cooled to room temperature and gently mixed manually with 500 µL of a solution composed of chloroform and isoamyl alcohol (24 parts chloroform to one–part isoamyl alcohol). Immediately after this, it was centrifuged at 5,000 g for 10 minutes to separate the organic phase from the aqueous phase.

As much of the supernatant solution as possible was recovered; this volume was then mixed with 0.6 volumes of isopropanol and centrifuged at 10.000 g for 20 minutes. The supernatant was discarded, and the precipitate was centrifuged at 10.000 g for 3 minutes with 300 µL of 70% ethanol. The resulting supernatant was discarded and the precipitate was dried at room temperature, leaving the tubes open for 24 hours. At the end of the protocol, a 50 µL solution of DNA diluted in TE buffer was obtained.

Polymerase chain reaction (PCR) was performed using the enzyme NZYTaq II DNA polymerase, supplied by NzyTech, following the protocol provided by the company. The PCR product was purified using ExoSAP–IT^TM^ supplied by Applied Biosystems. The samples were sent to the Gonçalo Moniz Institute – Fiocruz, Bahia (Brazil) for sequencing. The following primers and programs (Table 1) were used for DNA amplification and sequencing: ITS5/ITS4 (ITS nrDNA region), LR0R/LR5 (LSU nrDNA region) and *ATP6*–1/*ATP6*–2 initiators (*ATP6* mtDNA region).

**Table 1:** Primers utilized in this study and their respective sequences.

Primer	Type	Sequence	Program	Reference
ITS1	Nuclear	5’ TCCGTAGGTGAACCTGCGG 3’	95°C for 3 minutes (1x). 94°C for 30 seconds, 54° for 30 seconds, 72°C for 30 seconds (25x). 72°C for 5 minutes (1x). 4°C on infinite hold.	White et al. [24]
ITS4	Nuclear	5’ TCCTCCGCTTATTGATATGC 3’		White et al. [24]
LR5	Nuclear	5’ TCC TGA GGG AAA CTT CG 3’	94°C for 2 minutes (1x). 94°C for 30 seconds, 52°C for 30 seconds, 72°C for 1.5 minutes (33x). 72°C for 10 minutes (1x). 4°C on infinite hold.	Vilgalys [25]
LR0R	Nuclear	5’ ACC CGC TGA ACT TAA GC 3’		Vilgalys [25]
*ATP6*–1	Mitochondrial	5’ ATTAATTSWCCWTTAGAWCAATT 3’	95 °C for 2 minutes (1x). 94°C for 35 seconds, 37°C for 55 seconds (5x). 72°C for 1 minute (1x). 94°C for 35 seconds, 45°C for 55 seconds, 72°C for 1 minute (35x). 72°C for 5 minutes (1x). 4°C on infinite hold.	Kretzer & Bruns [26]
*ATP6*–2	Mitochondrial	5’ TAATTCTANWGCATCTTTAATRTA 3’		Kretzer & Bruns [26]

### Phylogenetic analyses

The consensus sequences were obtained and edited manually with the aid of Sequencher^TM^ version 4.1.4 software, using the “Dirty Data” algorithm to assemble the sequences, with 70 being the minimum value for the Minimum Match Percentage, and 20 as the minimum value for the Minimum Overlap. In addition to the sequences generated in this study, for a better composition of the molecular data matrix, sequences from *Itajahya* and *Phallus* deposited in GenBank were retrieved; all new sequences presented here were deposited in GenBank.

The alignment process was performed using MAFFT version 7 plataform, using the integrative refinement method E-INS-i. The alignments (ITS, LSU, and *atp6*) were submitted to jModelTest version 2.1.10 to choose the best substitution model using the Akaike Information Criterion, selecting 3 as the number of substitution schemes in the likelihood settings. The following models were selected: GTR + I + G for ITS and LSU, and GTR + G for *atp6*. The sequences were concatenated using Sequencer Matrix version 1.7.8 software.

The generated script was submitted to the CIPRES platform for Bayesian and Maximum Likelihood analysis, using the MrBayes on ACCESS (3.2.7a) tool, with 50.000.000 generations for the Bayesian Inference, and the RAxML-HPC BlackBox (8.2.12) tool with a partitioned model for obtain the Maximum Likelihood. The files containing the generated topology was viewed in FigTree (version 1.4.4) software. For the external group, sequences from the species *Mutinus albotruncatus* B.D.B. Silva & Baseia, *M*. *fleischeri* Penz., *M*. *taishanensis* P.M. Wang & Z.L. Zhou and *M*. *verrucosus* T.S. Cabral, B.D.B. Silva, K. Hosaka, M.P. Martín & Baseia were used (Table 2).

**Table 2:** Taxa used in Bayesian analysis and their respective accession numbers in GenBank.

Taxon	Voucher	ITS	nucLSU	*ATP6*
*Itajahya galericulata*	FURB 59764	PX921019	PX915467	PX925635
*I. galericulata*	FURB 59758	–	PX735362	–
*I. galericulata*	FURB 59746	–	PX735361	–
*I. galericulata*	PREM 61268^c^	–	KR071850	KR071847
*I. galericulata*	PREM 61269	–	KR071851	KR071848
*I. mucugensis* sp. nov. HOLOTYPE	ALCB133031	PX921020	PX915466	PX925637
*I. pakistanica* HOLOTYPE	LAH38385	PQ660738	–	–
*I. pakistanica*	LAH38386	PQ660739	–	–
*I. payaya* sp. nov. HOLOTYPE	ALCB141144	PX921018	–	PX925636
*I. punjabensis* HOLOTYPE	LAH38387	PQ660736	–	–
*I. punjabensis*	LAH38388	PQ660737	–	–
*I. rosea*	UFRN–Fungos1001	MG678532	–	MG678560
*I. rosea*	UFRN–Fungos535	–	JF968441	JF968439
*Mutinus albotruncatus*	UFRN–Fungos2025	KT202281	KT183493	KT183490
*M. fleischeri*	INPA240012	–	MW546299	–
*M. taishanensis*	EFHAAU2894	–	OP584494	OP612728
*M. verrucosus*	UFRN– Fungos 2026	NR_160483	MF447809	–
*Phallus denigricans* HOLOTYPE	INPA–Fungos 272383	MG678486	MG678455	MG678541
*P. indusiatus* NEOTYPE	INPA–Fungos 264931	MG678500	MG678463	MG678550
*P. purpurascens* HOLOTYPE	UFRN–Fungos 2808	MG678487	MG678456	MG678542
*P. purpurascens*	SINOP28	MG678495	MG678459	MG678545
*P. squamulosus*	UFRN–Fungos 2806	MG678497	–	MG678547
*P. cinnabarinus*	INPA–Fungos 255835	KJ764821	MG678471	MG678561
*P. cinnabarinus*	INPA–Fungos 255836	MG678533	MG678472	MG678562
*P. coronatus*	LE 295238	MG678522	MG678466	MG678554
*P. flavocostatus*	RE2004	MG678524	MG678467	MG678556
*P. dongsun* HOLOTYPE	GDGM 75402	MN307397	MN264679	–

### Integrative taxonomic protocol and species concept utilized

The new species proposed here follows the guidelines proposed by Aime et al [27]. In this study, two sets of data were analyzed: **i**. morphology, **ii**. molecular data. The morphological data set can be subdivided into **i**. macromorphological data and **ii**. micromorphological data. The molecular data correspond to three markers (two nuclear and one mitochondrial), analyzed here jointly. Both data sets were considered equally, assigning equal value to morphological features and molecular analyses. The data were integrated using the congruence integration protocol proposed by Padial et al [28]. In this work, the unified species concept proposed by de Queiroz et al. [29] was considered.

### Nomenclature

The electronic version of this article in Portable Document Format (PDF) in a work with an ISSN or ISBN will represent a published work according to the International Code of Nomenclature for algae, fungi, and plants, and hence the new names contained in the electronic publication of a PLOS article are effectively published under that Code from the electronic edition alone, so there is no longer any need to provide printed copies.

The new names contained in this work have been submitted to MycoBank from where they will be made available to the Global Names Index. The unique MycoBank number can be resolved and the associated information viewed through any standard web browser by appending the MycoBank number contained in this publication to the prefix http://www.mycobank.org/MB/. The online version of this work is archived and available from the following digital repository: PubMed Central, LOCKSS.

## Results

### Phylogenetic analyses

In this study ten new sequences were generated from the analyzed *Itajahya* specimens, including three ITS, four nucLSU, and three *atp6* sequences. Among these, there are four topotypes sequences of *I*. *galericulata* (specimens collected at the type locality). The concatenated matrix comprised 20 ITS, 20 nucLSU, and 19 *atp6* sequences, totaling 2163 positions (598 from ITS, 912from nucLSU, and 653 from *atp6*). The nucleotide substitution model using jModelTest for ITS and nucLSU was identified as GTR + I + G, and for *atp6* as GTR + G, used for the bayesian inference.

In the two analyses performed — Bayesian Inference and Maximum Likelihood — it was possible to recover the monophyly of the genus *Itajahya* (Fig 2). Phylogenetic analyses recovered *Itajahya mucugensis* and *I. payaya* as sister taxa, resolved within a single, well–defined clade. This relationship is supported by their close phylogenetic placement and by relative differences in branch lengths observed in the inferred tree topology.

**Fig 2:**
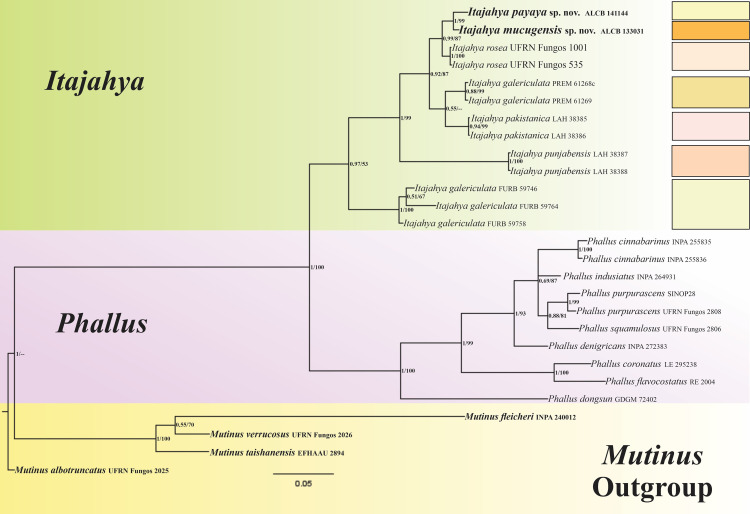
Bayesian consensus tree obtained from concatenated data from ITS, nucLSu, and *atp6* markers. Genera are highlighted in different colors, green for *Itajahya*, purple for *Phallus*, and yellow for *Mutinus*. The color in rectangles indicate the pseudoestipe color of taxa. New species are marked in bold. The values at the nodes indicate maximum likelihood/posterior probabilities.

*Itajahya mucugensis* and *I. payaya* formed a well–supported clade with *I. rosea* (IB = 0.99 MLbs = 87%). *Itajahya galericulata* from South Africa formed a clade distinct from the material from the type locality, Brazil. The sequences from South Africa correspond to different taxon than *I. galericulata*, indicating a closer relationship *I. pakistanica*.

No DNA sequences are currently available for *Itajahya argentina* and *I*. *hornseyi*. Both taxa are known exclusively from their original descriptions and are represented by single historical collections. They remain as obscure taxa, collected only once, with the only records for science being the respective works describing the species.

### Taxonomy

***Itajahya mucugensis*** M.S. Ribeiro, T.S. Cabral & B.D.B. Silva, sp. nov. (Figs 3–6.)

**Fig 3:**
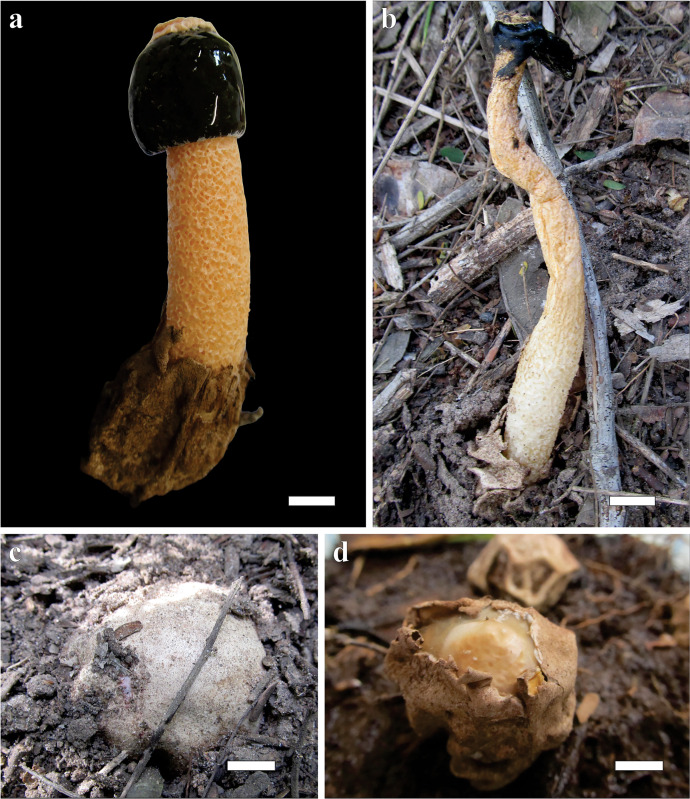
Itajahya mucugensis. **a.** Isotype UFRN Fungos 3819 (bar = 10 mm). **b.** Holotype ALCB 133031 in the field (bar = 10 mm). **c.** immature basidiome in the field (bar = 5 mm). e. immature during the initial breakup of the peridium.

**Fig 4:**
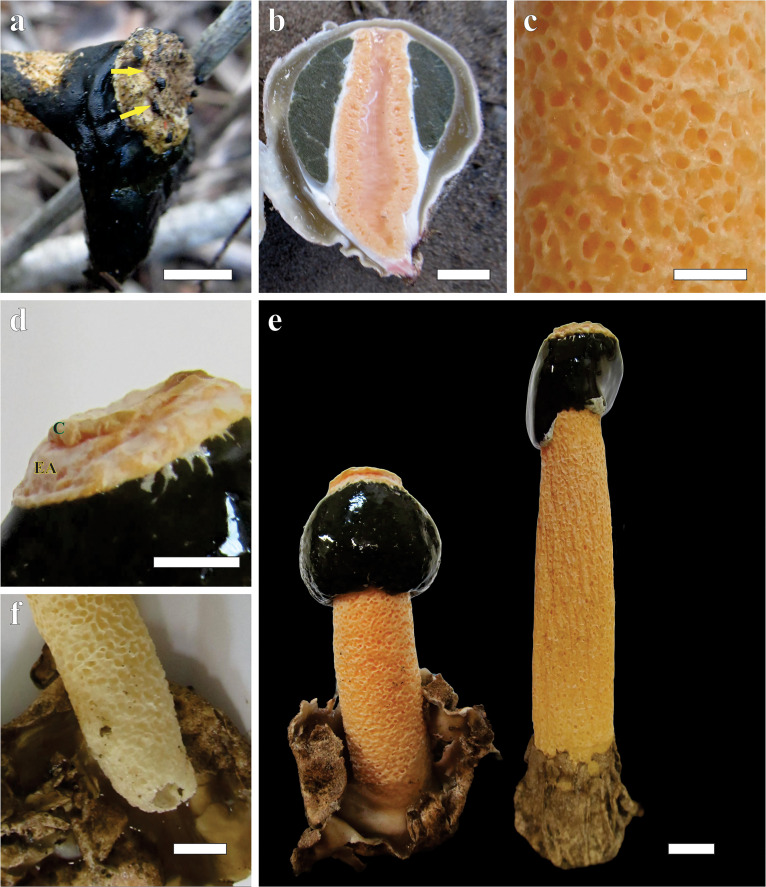
Details of *Itajahya mucugensis*. a. detail of expanded apex with calyptra from holotype (ALCB 133031) with arrows pointing to the edge of calyptra (bar = 5 mm). **b.** Longitudinal section of the immature basidiome (bar = 5 mm). **c.** Detail of the pseudostipe (bar = 5 mm). **d.** detail of expanded apex with calyptra from isotype (UFRN Fungos 3819), EA = expanded apex, C = Calyptra (bar = 5 mm). **e.** Isotypes, basidiome newly expanded (left), fully expanded (right), (bar = 10 mm). **f.** Detail of the base of the pseudostipe (bar = 10 mm).

**Fig 5:**
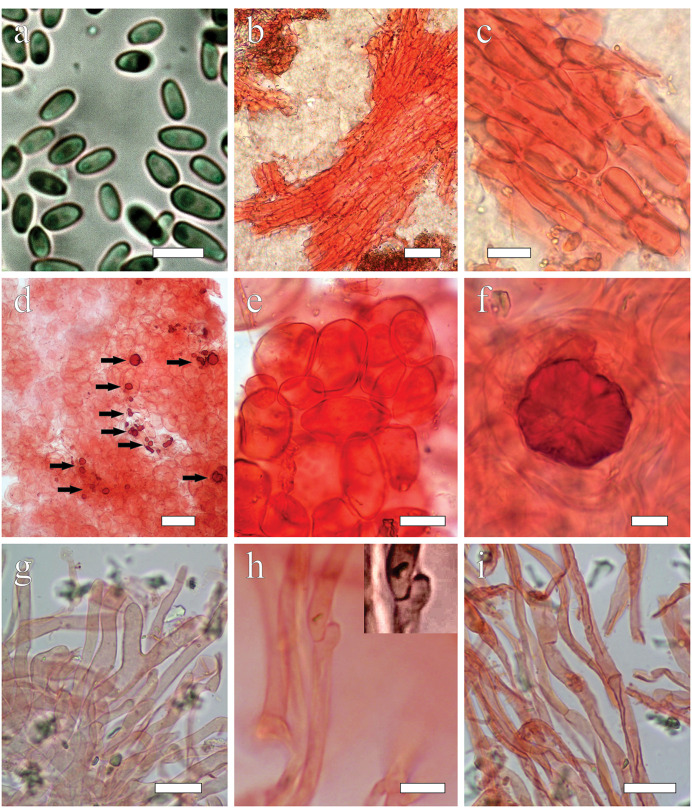
Microscopy of *Itajahya mucugensis* from holotype and isotype. **a.** Basidiospores in 5% KOH (bar = 5 μm). **b.** Organization of hyphae in the receptacle (bar = 100 μm). **c.** Hyphae in the receptacle (bar = 20 μm). **d.** Hyphae of the pseudostipe; arrows indicate crystals (bar = 50 μm). **e.** Detail of the hyphae of the pseudostipe (bar = 20 μm). **f.** Detail of a crystal (bar = 10 μm). **g.** Hyphae of the exoperidium (bar = 20 μm). **h.** Mesoperidium hyphae and detail of a connecting clamp (bar = 10). **i.** Endoperidium hyphae (bar = 20 μm).

**Fig 6:**
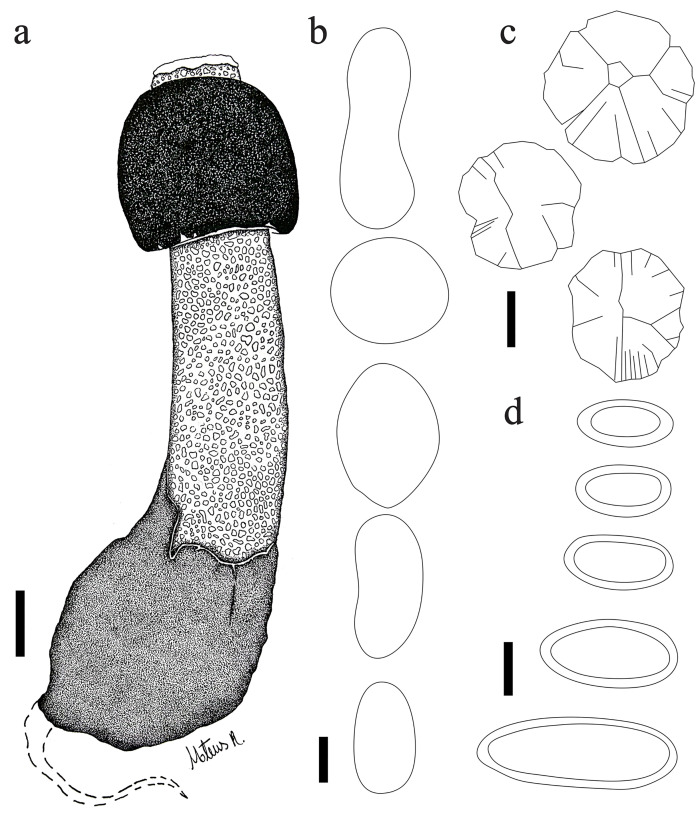
Drawings of *Itajahya mucugensis.* **a.** Scientific illustration of the holotype (bar = 10 mm). **b.** Calyptra cells (bar = 10 μm). **c.** Schematic representation of crystals found in the volva and pseudostipe (bar = 5 μm). **d.** Basidiospores (bar = 2 μm). Made by the first author.

**Mycobank**: #861701

**Diagnosis**: This species is characterized by the bright orange color of its pseudostipe, observed in both immature and mature basidiomata, presence of crystals in the mesoperidium and at the base of the pseudostipe

**Holotype**: Brazil, Bahia, Mucugê: 41°00’34”S 41º23’45”W (approximate coordinates) on sand soil, B.D.B Silva, M.S. Ribeiro, R.R. Fermiano, R.V.B. Araújo, (ALCB133031!), ITS, nucLSU and *atp6* sequences (PX921020, PX915466, PX925637). **Isotype**: Brazil, Bahia, Mucugê: 41°00’34”S 41º23’45”W (approximate coordinates) on sand soil, B.D.B Silva, M.S. Ribeiro, R.R. Fermiano, R.V.B. Araújo (UFRN Fungos 3819).

**Etymology**: The name refers to the municipality of Mucugê, city localized in Chapada Diamantina Complex, in the Bahia state, Brazil, where the specimens were collected.

**Macromorphology**: Immature basidiomata hypogeous, subglobose to globose, grayish (N_00_Y_00_M_00_ – N_10_Y_00_M_00_), measuring 36 mm high × 20 mm in diameter (dry). Expanded basidiomata 85–135.8 mm high. Volva composed of three layers, persistent, brown (N_50_Y_50_M_40_ – N_50_Y_60_M_50_), measuring 35–36 high ×24–26 mm in diameter. Exoperidium grayish white when fresh, turning brown (concolor with volva) in immature basidiomata detached from the ground, fleshy, dry at touch. Mesoperidium translucent, gelatinous in appearance, moist to the touch when fresh. Endoperidium white (N_00_M_00_Y_00_), dry at touch. Pseudoestipe firm, spongey, hollow, orange in newly expanded basidiomata, becoming pale with age (N_00_Y_30_M_00_ – N_00_Y_40_M_00_ – N_00_Y_50_M_00_), whitish near the base, with an expanded apex, 66–74.9 mm high × 17–18.2 mm diameter in totality. This orange color is also seen on the pseudostipe of immature specimens. Receptacle subapical, white (Y_00_M_00_C_00_) formed by a layer that hangs from the apex and expands vertically. From this layer, white hyphae laminae expand, supporting the gleba, measuring 14–20 mm high ×19–25 mm diameter. Gleba greenish black (N_99_C_50_Y_99_), mucilaginous. Calyptra orange (concolor with pseudoestipe), 9 mm in diameter.

**Micromorphology**: Rhizomorph not observed. Exoperidium composed of filamentous hyphae, hyaline, some branched, septate, clamp connections not observed, 4.4–23.7μm width. Mesoperidium composed of filamentous hyphae, septate, some with inflated tips, clamp connections present, 2.7–9.8 μm width. Endoperidium composed of filamentous hyphae, hyaline, some septate, rare ramifications, 1.4–7.2 μm width. Pseudoestipe composed of subglobose to ellipsoid hyphae, organized in layers of pseudoparenchymatose aspect, hyalines, showing no significant morphological differences between the strata analyzed, 20.8–63 μm length ×13–51.1 μm width. Receptacle composed of elongated and robust hyphae, with a few globose cells, hyalines, 20–92.3 μm length ×8.4–21.8 μm width. Gleba composed of basidiospores at maturity. Calyptra composed of hyphae ranging from subglobose to elongated, hyaline, 18–59.8 μm length × 12–28.5 μm width. Basidia not observed. Basidiospores cylindrical, smooth, 3.6–7.3 μm length × 1.8–3 μm width, greenish in 5% KOH, walls 0.2–0.5 μm width. Rosette–shaped crystals contained within globular cells, observed in the mesoperidium and between the cells at the base of the pseudostipe, measuring 10–24.9 μm length × 9.8–22.7 μm width in mesoperidium and 22.6–41.9 μm length ×20.7–37.7 μm width in pseudoestipe.

**Habitat**: Solitary basidiomata, growing on sand soil, shaded environment.

**Note**: Previously registered as *Itajahya galericulata* in the state of Bahia [13], morphological and molecular analyses revealed that the specimens presented in this study belong to a distinct taxon. This species is distinguished from all other *Itajahya* species by its bright orange basidiome. *Itajahya galericulata* and *I*. *argentina* has a white pseudostipe [1]; *I*. *pakistanica*, *I*. *punjabensis* and *I*. *rosea* has a pink one [2–4,7]. *Itajahya hornseyi* has a yellowish to slight pink one [5]. When compared to *Itajahya payaya* sp. nov., in addition to its orange pseudostipe (yellowish white in *I*. *payaya*), *I*. *mucugensis* presents differences in its micromorphology due to the presence of crystals in the mesoperidium and at the base of the pseudostipe, while these are completely absent in *I*. *payaya*.

***Itajahya payaya*** M.S. Ribeiro, T.S. Cabral & B.D.B. Silva, sp. nov. (Figs 7–10)

**Fig 7:**
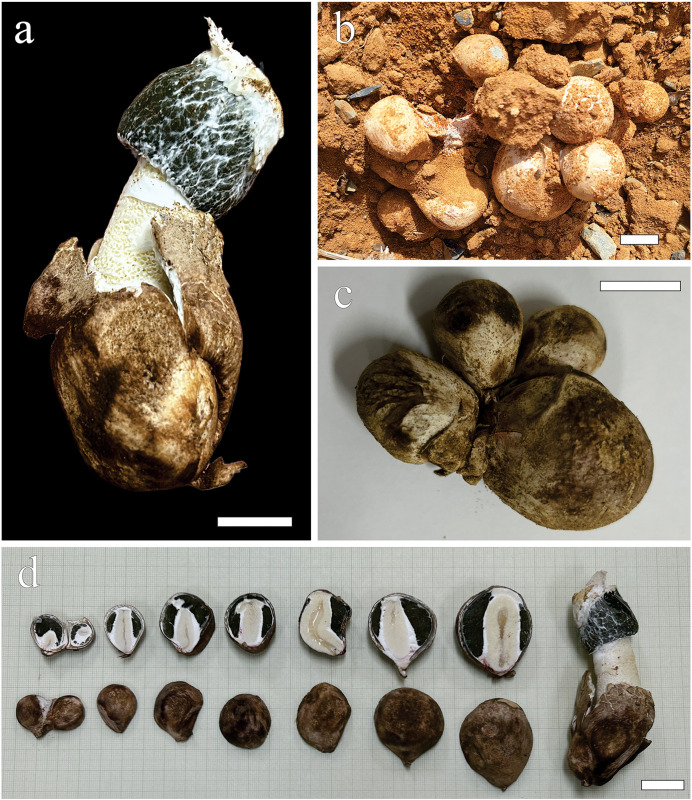
Itajahya payaya. **a.** expanded basidiome of *Itajahya payaya* holotype (ALCB 141144) (bar = 20 mm), **b.** Immature basidiomata growing on soil (bar = 20 mm), **c.** detail of the darkening of the peridium of immature basidiomata after detachment from substrate (bar = 20 mm), **d.** immature sectioned basidiomata and holotype showing stages of development. Photos: a, c, d – Mateus S. Ribeiro, b – Filipe S. Batista.

**Fig 8:**
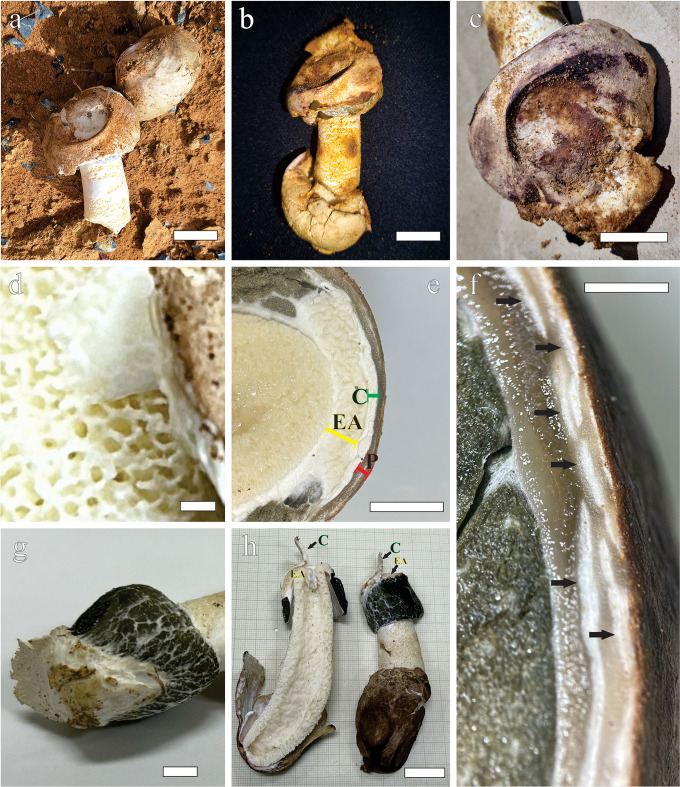
Details of *Itajahya payaya.* **a.** immature basidiome and broken basidioma (isotype ALCB141164) with part of the peridium covering the receptacle (bar = 20 mm), **b.** expanded basidiome from isotype ALCB141164 (bar = 20 mm), **c.** detail of the peridium from isotype ALCB141164, showing purple reaction in lacerated areas (bar = 20 mm), **d.** detail of pseudoestipe from holotype (bar = 2 mm), **e.** section of an immature basidiome, showing the expanded apex (EA), calyptra (C), and peridium (P) (bar = 10 mm), **f.** cross–section showing the layers of the peridium and details of the intrusions of white hyphal plates (arrows) (bar = 20 mm), **g.** detail of calyptra (bar = 20 mm), **h.** section of the holotype showing the inner part of the basidioma (bar = 20 mm). Photos: a, b, c – Filipe S. Batista, d, e, f, g, h – Mateus S. Ribeiro.

**Fig 9:**
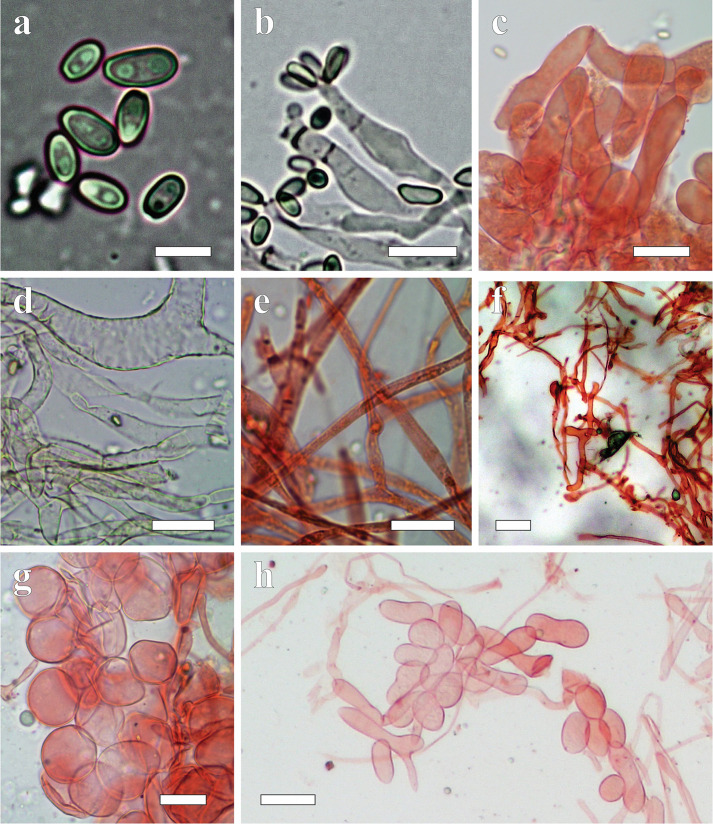
Microscopy of *Itajahya payaya* from holotype. **a.** Basidiospores in 5% KOH (bar = 5 μm). **b.** Basidia (bar = 10 μm). **c.** Receptacle hyphae (bar = 20 μm). **d.** Exoperidium hyphae (bar = 20 μm). **e.** Mesoperidium hyphae (bar = 20 μm). **f.** Endoperidium hyphae (bar = 30 μm). **g.** Pseudostipe hyphae (bar = 20 μm). h. Calyptra hyphae (bar = 30 μm).

**Fig 10:**
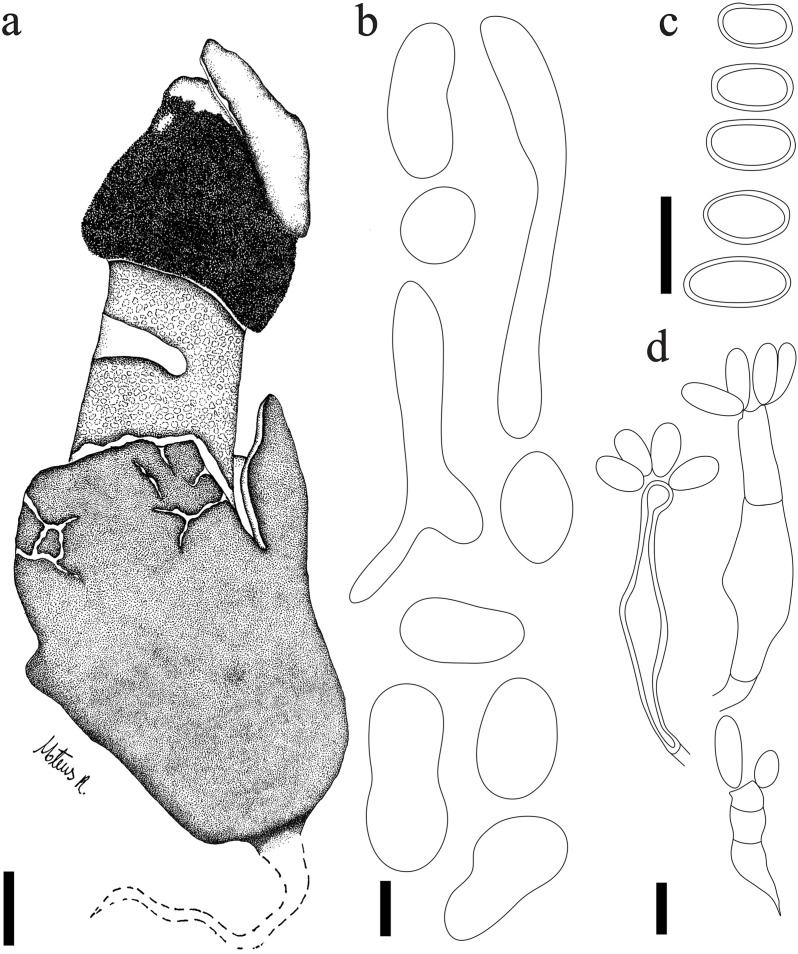
Drawings of *Itajahya payaya.* **a.** Holotype (bar = 10 mm). **b.** Calyptra hyphae (bar = 10 μm). **c.** Basidiospores (bar = 5 μm). d. Basidia (bar = 5 μm). Made by the first author.

**Mycobank**: #861702

**Diagnosis**: Basidiospores measuring 3.9–6.4 μm length × 2.4–3 μm width, pseudoestipe yellowish white, campanulate receptacle and mesoperidium with intrusions of white hyphal plates.

**Holotype**: Brazil, Bahia, Morro do Chapéu: Canabravinha, 11°36’49.7”S 41°14’05.0”W, 08 Jun. 2024, leg. Batista, F.S. & Nascimento, L.A.G. (ALCB141144!), ITS, *atp6* Genbank sequences: PX921018, PX925636). **Isotype**: Brazil, Bahia, Morro do Chapéu: Canabravinha, 11°36’49.7”S 41°14’05.0”W, 08 Jun. 2024, leg. Batista, F.S. & Nascimento, L.A.G. (UFRN Fungos 3820). **Isotype**: Brazil, Bahia, Morro do Chapéu: Canabravinha, 11°36’49.7”S 41°14’05.0”W, 08 Jun. 2024, leg. Batista, F.S. & Nascimento, L.A.G. (ALCB141164).

**Etymology**: The name refers to the Payaya people, an indigenous community native to the Morro do Chapéu, city localized in Chapada Diamantina Complex, in the Bahia state, Brazil.

**Macromorphology**: Immature basidiomata subglobose, gregarious, measuring 19–42 mm high ×19–39 mm diameter, pale brown, showing a reddening to purple reaction when lacerated. Peridium composed of three layers, up to 2 mm thick in totality. Expanded basidiomata 100 mm high. Volva composed of three layers, persistent, brown (N_40_Y_40_M_30_ – N_50_Y_60_M_40_) grayish when dry (N_10_C_00_Y_00_), measuring 51.5 mm high × 31 mm diameter. Exoperidium grayish whitein the field (N_10_C_00_Y_00_), turning brown in immature basidiomata detached from the ground after a few hours, coriaceous, dry at touch. Mesoperidium translucent, gelatinous in appearance, moist to the touch when fresh, with intrusions of discontinuous layers of white hyphae; becomes cartilaginous in texture in basidiomata preserved in alcohol. Endoperidium white, thin, papyraceous, dry at touch. Pseudoestipe firm, spongey, hollow, yellowish white (N_00_Y_10_M_00_), with an expanded apex, 101 mm high, thicker in the lower half, ranging from 17–21 mm in diameter, 17 mm in diameter at the top, with the expanded apex measuring 21 mm in diameter, 10 mm high in totality. Receptacle subapical, formed by a white layer that hangs from the apex and expands vertically. From this layer, white hyphae laminae expand, supporting the gleba, measuring 23 mm high ×30 mm diameter.Gleba greyish green (N_60_C_20_Y_70_) in newly expanded basidiomata, dry at touch, cover with several white hyphal filaments, becoming mucilaginous and olive green (N_50_C_00_Y_90_ – N_50_C_00_Y_99_) with age. Calyptra yellowish white (concolor with pseudoestipe), 18.5 mm in diameter, 1.5–2.5 mm thick.

**Micromorphology**: Rhizomorph not observed. Exoperidium composed of filamentous hyphae, hyaline, some branched, septate, clamp connections not observed, 2.8–20.4 μm width. Mesoperidium composed of filamentous hyphae, septate, some with inflated tips, clamp connections present, 2.7–11 μm width. Endoperidium composed of filamentous hyphae, hyaline, 1.8–11.9 μm width. Pseudoestipe composed of subglobose to ellipsoid hyphae, organized in layers of pseudoparenchymatose aspect, hyalines, showing no significant morphological differences between the strata analyzed, 18.1–50.9 μm length ×12.5–37.9 μm width. Receptacle composed of elongated and robust hyphae, hyalines, 15.7–83.8 μm length ×11.3–18.8 μm width. Gleba composed of basidia, filamentous hyphae and basidiospores when immature, and basidiospores at maturity, and may present rare collapsed basidia in the newly expanded basidiome. Calyptra composed of hyphae ranging from subglobose to elongated, hyaline, 21.8–53.8 μm length × 10.9–22.6 μm width. Basidia present in immature basidiomata, degenerating into expanded basidiomata. Elongated basidia, hyaline, often thickened in the middle, 17.1–42.3 μm length × 3–6.6 μm width, some with septa, some with two septa, number of spores varying from two to six. Basidiospores elongated, smooth, 3.9–6.4 μm length × 2.4–3 μm width, greenish in 5% KOH, walls 0.2–0.4 μm width. Crystals not observed in any part or stage of basidiomata.

**Habitat**: Gregarious basidiomata, growing on soil, totally exposed to sunlight.

**Note**: *Itajahya payaya* is morphologically similar to *I*. *galericulata* and *I*. *argentina* due to its pale cream–colored pseudostipe. However, in addition to the molecular difference between *I*. *payaya* and *I*. *galericulata*, when observing the illustrations of the micromorphology of the latter in Möller’s work [1], it’s possible to note the absence of these white hyphal plate incrustations in the mesoperidium, present in *I*. *payaya* While *I*. *argentina* are differentiated by his small basidiomata, 65–70 mm, and larger basidiospores, 5–6 μm [2]. *Itajahya hornseyi*, *I*. *mucugensis*, *I*. *pakistanica*, *I*. *punjabensis* and *I*. *rosea* can be distinguished from *I*. *payaya* sp. nov. because none of the above species has a pale cream pseudoestipe [3,4,7].

## Discussion

The Chapada Diamantina region is characterized by high levels of endemism and a complex mosaic of vegetation formations. However, this region has historically been subjected to intense processes of degradation and transformation [30,31] making it a priority area for scientific research and conservation efforts. In this context, the results of this work highlight a diversity of fungal species that is still underestimated, reinforcing the region’s relevance for biodiversity studies.

In this context, the results of this study highlight a diversity of gasteroid fungi that has been underestimated, reinforcing the region’s importance for biodiversity studies. Furthermore, the sequences generated expand the molecular dataset currently available for *Itajahya*, including the first sequences of *I*. *galericulata* from the type locality, representing an important contribution to future taxonomic and phylogenetic studies on the genus.

Phylogenetic reconstruction using a concatenated ITS, LSU and *atp6* dataset supported the monophyly of *Itajahya*, clearly separating it from *Phallus*. The Bayesian and Maximum Likelihood analysis recovered independent and strongly supported clades corresponding to the newly described species, *I. mucugensis* and *I. payaya*. Despite their phylogenetic proximity, *Itajahya mucugensis* and *I. payaya* are morphologically distinct species, presenting striking differences such as the color of the pseudostipe and the nature of the peridium.

These clades were consistently distinct from *I. galericulata* and *I. rosea*, corroborating the morphological evidence that the specimens collected in the Chapada Diamantina do not belong to any previously described taxon. The close phylogenetic affinity between *Itajahya mucugensis* and *I. payaya* suggests a recent evolutionary history. The geographic proximity of their occurrences within the Chapada Diamantina region may have contributed to this pattern, reflecting shared ecological conditions or historical biogeographic processes that promoted lineage diversification in this area.

Notably, the two collection sites differ in phytosociological structure and environmental conditions, corresponding to arboreal-arbustive caatinga and rupestrian field vegetation, which may also have influenced the diversification of the lineages. Such ecological differences warrant further investigation in subsequent studies focused on habitat preferences and co-occurrence with specific plant communities.

To date, studies on plant-associated *Itajahya* species are scarce. *Itajahya galericulata* has been reported to be associated with *Jacaranda mimosifolia* in South Africa [12]. However, the molecular analyses presented in this study demonstrate that the individuals from South Africa correspond to a different species than *Itajahya galericulata*. Although the aforementioned studie use the term “association”, the specificity of this co-occurrence is unknown. While no direct evidence of plant interactions involving *Itajahya* species has been documented in Brazil, the records presented here reinforce the possibility of ecological associations with members of the local flora. It should be noted that *Jacaranda mimosifolia* does not occur in Bahia, the region where the specimens analyzed in this study were collected (https://floradobrasil.jbrj.gov.br).

However, *Jacaranda* species are widely distributed and frequently recorded in Bahia. These observations may indicate that *Itajahya* species are not necessarily associated with specific plant species, but perhaps with ecological conditions. Further studies are needed to clarify whether these occurrences represent specific ecological interactions, associations, or simply habitat preference.

The phylogenetic analyses presented in this study indicated that the current circumscription of *Itajahya galericulata* requires reassessment, as sequences derived from Brazilian and South African [12] specimens exhibit clear genetic divergence (Fig 2). This may indicate that the species *I. galericulata* is endemic to South America [32].

In fungal taxonomy, species descriptions based on multiple collections are generally preferred; however, this criterion cannot always be fully met in the case of rare taxa, taxa from highly specialized niches or remote locales, or those characterized by ephemeral basidiomata. Under such circumstances, the adoption of an integrative taxonomic approach, combining independent morphological and molecular datasets, provides a reliable framework for species recognition [27,33].

*Itajahya mucugensis* and *I. payaya* are proposed in this work from a single collection each (three mature basidiomata) but exhibit a unique combination of macro– and micromorphological characteristics, corroborated by their phylogenetic positioning as a distinct and well–supported lineage. These evidence, taken together, support their recognition as new species, despite the limited number of collections available.

The description of new fungal taxa based on a single or few collections is recurrent within the order Phallales, where several species have been proposed and validated in comparable situations, as also seen by Cabral and colleagues [33] within the order Agaricales, specifically in the genus *Tulostoma*. Among the representatives of Phallales, we can exemplify *Abrachium floriforme* (Baseia & Calonge) Baseia & T.S. Cabral [7], *Blumenavia baturitensis* Melanda, M.P. Martín & Baseia [20], *Clathrus cristatus* Fazolino, Calonge & Baseia [34], *Mutinus albotruncatus* B.D.B. Silva & Baseia [35], *Phallus calongei* G. Moreno & Khalid [36], and *Phallus squamulosus* T.S. Cabral, B.D.B. Silva & Baseia [18].

Therefore, the taxonomic decision presented here when evaluated under the perspective of integrative taxonomy, particularly the congruence integration protocol proposed by Padial et al. [28], the concordance between morphological and molecular data provides robust support for the delimitation and descriptions of two distinct taxa.

As a result of the taxonomic advances presented here, the genus *Itajahya* currently comprises eight species worldwide: *Itajahya argentina, I*. *hornseyi*, *I*. *galericulata*, *I*. *mucugensis*, *I*. *pakistanica*, *I*. *payaya*, *I*. *punjabensis* and *I*. *rosea.*

## Supporting information

S1 File lsu atp6 its concatenated. (FAS)
